# Transdermal Delivery of Ibuprofen Utilizing a Novel Solvent-Free Pressure-sensitive Adhesive (PSA): TEPI® Technology

**DOI:** 10.1007/s12247-017-9305-x

**Published:** 2017-12-14

**Authors:** Emma L. Tombs, Vasiliki Nikolaou, Gabit Nurumbetov, David M. Haddleton

**Affiliations:** 1 Medherant Ltd, The Venture Centre University of Warwick Science Park, Coventry, CV4 7EZ UK; 2 Chemistry Department, University of Warwick, Library road, Coventry, CV4 7AL UK

**Keywords:** Ibuprofen, PSA adhesive, Solvent-free system, Drug delivery, Transdermal patch

## Abstract

**Purpose:**

The main objective of this present study was the investigation of potential novel transdermal patch technology (TEPI®) delivering ibuprofen as the active pharmaceutical ingredient (API) using a novel poly(ether-urethane)-silicone crosslinked pressure-sensitive adhesive (PSA) as the drug reservoir in a solvent-free manufacturing process.

**Methods:**

The patch was synthesized utilizing the hot-melt crosslinking technique without the addition of solvents at 80 °C in 100% relative humidity. Dissolution and permeation studies performed utilizing diffusion cells and subsequently HPLC validated methods were employed to determine the API content in the acceptor solution. Accelerated stability studies were also performed at 40 °C and 70% relative humidity. The adhesive performance of the fabricated patch was evaluated utilizing loop tack adhesion tests.

**Results:**

In vitro permeation experiments across both Strat-M® and human skin demonstrated that ibuprofen can easily be released from the adhesive matrix and penetrate through the studied membrane. A comparison on the permeation rates of the API across the two membranes indicated that there is not a strong correlation between the obtained data. The presence of chemical enhancers facilitated an increased flux of the API higher than observed in the basic formulation. Initial stability studies of the optimized formulation showed no degradation with respect to the drug content. Adhesion studies were also performed indicating higher values when compared with commercially available products.

**Conclusions:**

The present study demonstrated the fabrication of an ibuprofen patch utilizing a versatile, solvent-free drug delivery platform. Upon optimization of the final system, the resulting patch offers many advantages compared to commercially available formulations including high drug loading (up to 25 wt%), good adhesion, and painless removal leaving no residues on the skin. This PSA offers many advantages over existing adhesive technology.

**Graphical Abstract:**
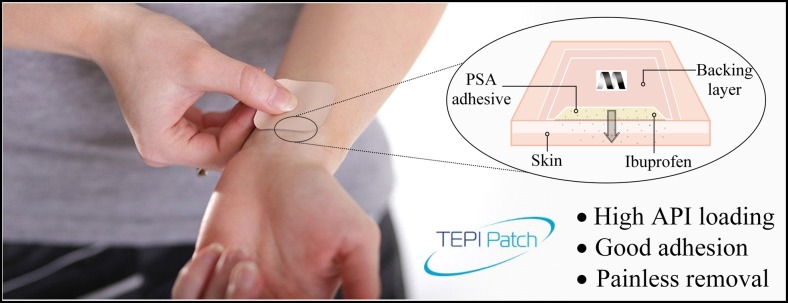
ᅟ

**Electronic supplementary material:**

The online version of this article (10.1007/s12247-017-9305-x) contains supplementary material, which is available to authorized users.

## Introduction

Transdermal drug delivery was first introduced commercially in 1979 with the scopolamine patch, and since then, it has been an attractive route for drug delivery as well as a challenging area of research. Topical delivery offers compelling advantages in comparison with more conventional delivery systems as it provides many advantages over conventional routes by enhancing patient compliance, avoiding first-pass metabolism, and minimizing harmful side effects due to overdose [1]. Additionally, it can address the limited controlled release and the low bioavailability of many oral drugs avoiding a potential damage to the gastrointestinal tract. Transdermal delivery systems can also be preferred as they are non-invasive, self-administered, and generally inexpensive [2–4]. Hence, over the past few years, many drugs (including nicotine, lidocaine, methyl salicylate, hormones, and fentanyl) have been utilized for the development of transdermal patches to treat various conditions.

A main disadvantage of drug delivery patches is that they are frequently unable to convey the desired active ingredient through the skin, as it is well established that the major penetration barrier resides within the stratum corneum [5–7]. Its complex structure forces the permeant to travel between multiple hydrophilic and hydrophobic domains which is unfavorable in most cases [8]. Consequently, the drug employed should be relatively both hydrophilic and hydrophobic to successfully penetrate the skin. Non-steroidal anti-inflammatory drugs (NSAIDs) are good candidates as they often have low molecular weights with appropriate physicochemical properties and their transdermal delivery has already been reported [9–11]. Ibuprofen is one of the most prominent drug among NSAIDs and is routinely employed to ease moderate pain, reduce swelling, control fever, and treat arthritis at higher doses [12–15]. However, along with most of other NSAIDs, ibuprofen has risks and complications including inflammation of the stomach (gastritis) which may result in a stomach ulcer or even bleeding [16, 17].

Transdermal systems can modulate the drug release levels on site for long periods of time but the absolute amount permeating the skin is strongly dependent upon the matrix in which the active drug is applied. Various polymers are routinely employed as the adhesive matrix including cellulose derivatives, chitosan, polyacrylates, polyurethanes, and silicone-based polymers [18–20]. Regardless its chemical nature, the adhesive must cause no skin irritation and sensitization during skin contact, provide sufficient adhesion to ensure optimum dosage, and be easily removed without leaving any residues on the skin. Among the available systems, silicon-based pressure-sensitive adhesives (PSAs) are of interest mainly due to their low irritation, good skin adhesion, and favorable diffusion characteristics for many APIs [21–23]. However, typical PSA systems employ volatile organic solvents (VOCs) raising environmental and financial concerns. Stemming from these concerns, various solvent-free delivery systems have been introduced to eliminate VOC emissions and possibly provide properties to extend the use of these materials to new applications [23]. Hot-melt silicone-based PSAs (HMPSAs) offer many advantages as they are less expensive, they can be softened to viscosities suitable for coating and then return to a flawless state upon cooling. Moreover, they can be formulated to contain little or no chemical functionality reducing the possibility of interactions with the API [24] making these systems a very attractive option for medical applications.

In this present study, a novel TEPI® transdermal patch technology is reported and is exemplified with ibuprofen as the active pharmaceutical ingredient (API) and a new HMPSA crosslinked polymer excipient as a drug reservoir. No volatile solvents are employed in any stage of the patch production with all added components present in the final device. The API release from the new PSA was studied employing non-rate-limiting membranes. Both synthetic [25, 26] and natural membranes were employed to assess permeability of the API and in vivo performance over a 24-h period employing diffusion cells. A brief comparison between these membranes was also reported. Chemical enhancers (low molecular weight excipients) were added in to the formulation to increase skin permeability enabling molecular transport of the API across human skin membranes [8, 27–29]. The final, optimized system offers many advantages compared to commercially available formulations including high loading, good adhesion. Finally, accelerated stability studies at 40 °C and 70% relative humidity suggested that the optimized formulation is stable with respect to its drug content, adhesion, and physical characteristics.

## Materials and Methods

### Materials

All materials were used as received unless otherwise stated. Ibuprofen (Ph. Eur. S380) was purchased from Shasun Pharmaceuticals (Chennai, India). The pre-cured adhesive matrix utilized in all the patches was provided by Bostik (Cedex, France). Titanium n-butoxide, benzyl alcohol (BA), di(ethylene) glycol monoethyl ether (DEGEE), propylene glycol (PG), polyethylene glycol 400 (PEG_400_), oleic acid (OA), octadecanol (OD) phosphate buffer saline (PBS, pH 7.4), and trifluoroacetic acid (TFA) were purchased from Sigma-Aldrich in the highest available grade. HPLC gradient grade water and acetonitrile (MeCN) were also purchased from Sigma-Aldrich. Strat-M® membrane was purchased from Merck Millipore. Nylon (0.45 μm pore size) membrane was purchased from Fisher Scientific. Human male or female abdominal skin samples (400 ± 50 μm) were sourced fresh from a licensed company in strict accordance with the Human Tissue Act 2004 and Human Tissue Regulations 2007 and codes of practice.

### Instrumentation

The HPLC (Agilent 1220 Infinity LC equipped with variable wavelength detector) was fitted with a Phenomenex Kinetex® EVO column, 5 μm C18 (l = 50 mm; ∅ = 4.6 mm) with Security Guard ULTRA guard cartridge (P/N AJ0-9296). Patches were fabricated using a coater purchased from RK Print Coat Instruments Ltd. (K Control Coater K101). The coater is equipped with a heated bed and a bar coating head which uses a push bar to drive a gap applicator along the substrate being coated. A micrometer adjustable spreading blade was used to accurately set the substrate/blade gap up to 10,000 μm in 10 μm increments.

### HPLC Validation

A validated reverse phase HPLC methodology was used to determine the content of the API in the acceptor medium. Detection was achieved using a spectrophotometer at λ = 264 nm. The flow rate of the eluent was set to 1.0 mL min^−1^ and all injections of 50 μl. Data was collected and analyzed using Agilent Technologies OpenLAB CDS software (version C.01.07 [27]). Mobile phase solvents used were HPLC grade and consisted of mobile phase (a) 100% *v*/*v* water, 0.1% TFA; mobile phase (b) 100% *v*/*v* MeCN. The calibration curve for ibuprofen was linear at concentrations ranging from 1 to 50 μg mL^−1^. The accuracy of the assay was tested by triplicate injection of five samples within 50–200% range of nominal concentration (50 μg mL^−1^), and subsequent calculation of the recovery percent. Estimated average values of recovery and RSD were found to meet accuracy criteria of 100 ± 2% and < 1%, respectively. The limit of detection (LOD) was defined as lowest amount of ibuprofen which can be reproducibly detected as the signal which is three times higher than the baseline and was found to be 200 ng mL^−1^. The limit of quantification (LOQ) was defined as the lowest amount of analyte which reproducibly gives signal-to-noise ratio (SNR) of 10/1 and was found to be 600 ng mL^−1^.

### Ibuprofen Patch Preparation and Curing Process

All formulations were prepared using mass fractions of ingredients. Initial experiments employed the basic formulation (API, curing catalyst, and benzyl alcohol) without the addition of any penetration enhancers. A short optimization excipient study resulted in the optimized formulation given in Table 1. The adhesive matrix was charged in a reactor equipped with an overhead stirrer and was heated to 80 °C under inert atmosphere. Subsequently, a mixture of the curing catalyst, benzyl alcohol, and the permeation enhancers was added in the reactor and the mixture was left to stir at 300 rpm for 5 min. Ibuprofen and oleic acid were added next and the mixture was left to stir for further 5 min before it was cast on a release liner and spread uniformly utilizing an automated film coater. The resulting film was subsequently heated in an oven at 75 °C in 100% humid atmosphere for 4 min. A backing layer was added onto the surface upon completion of the curing.

**Table 1:** List of components used for the synthesis of the optimized formulation for the ibuprofen patch

Ingredients	Function	Mass fraction, wt%
Adhesive	Adhesive matrix	75.00
Ibuprofen	API	10.00
Titanium n-butoxide	Curing catalyst	1.50
Benzyl alcohol	Co-solvent	2.00
DEGEE	Permeation enhancer	5.00
PG		5.00
Oleic acid		1.50

### Drug Content/Extraction

Discs were cut from the test patch using a die cut punch, 8 mm (0.50 cm^2^) and the weight recorded. Upon removal of the release liner, the disc was placed into a vial equipped with a stirrer and 10 mL of MeCN:H_2_O (50:50 *v*/*v*) solution was added. The mixture was left for 24 h at 60 rpm. Using a syringe, approximately 1 mL was extracted from the sample vial and dispensed through a filter into a 2 mL HPLC vial. A validated HPLC method was then used to determine the ibuprofen content. The above procedure was repeated in 12 replicates to ensure repeatability of the results.

### Permeation and Release Studies

Samples were cut to size and applied to the membranes (Strat-M and human skin were used for permeation, nylon was used for release experiments) at ambient temperature. Upon application, patches were placed into jacketed diffusion cells (A = 1 cm^2^). Prior to the start of the experiment, the acceptor solution (PBS:Transcutol, 90:10 *v*/*v*) was equilibrated at 32 ± 1 °C for 15 min by maintaining the jacket temperature at 36 °C. The acceptor medium was stirred at 600 rpm throughout the experiment. Samples were taken at regular intervals and analyzed using the HPLC validated method. Six diffusion cells were employed in parallel for each formulation. Reported cumulative amounts and flux values represent the mean of six measurements (*n* = 6).

### Adhesion Studies

The adhesion of patches was determined using a Mecmesin MultiTest 2.5-d+ testing system equipped with an Advanced Force Gauge 50N. The instrument was set up in accordance with the manufacturer’s guidelines to perform loop tack tests. Samples were cut to 175 × 25 mm. Stainless steel was employed as the substrate. The maximum peak force (N) required to detach a patch from the substrate was taken as the evaluation parameter. An average of three measurements (*n* = 3) was accepted as a statistically robust value of adhesion.

### Stability Studies

Short-term stability studies of the optimized formulation performed at 40 °C and 70% relative humidity to confirm the stability of the API in the formulation. Parameters determined during the stability study included extraction studies and visual inspection. The patches were stored in aluminum pouches prior to testing. Samples were withdrawn periodically and evaluated for the different points of analysis utilizing HPLC validated methods and visual inspection.

## Results and Discussion

### Compatibility of Ibuprofen with the TEPI® System

The pressure-sensitive adhesive (PSA) employed consists of a proprietary poly(propylene glycol)-based polyurethane (PPG-PU) with trimethoxysilane (SiOMe_3_) terminal functional groups (a silylated polyurethane, SPUR). Upon contact with moisture, SiOMe_3_ undergoes a condensation (curing) reaction to form a crosslinked polymer matrix with the release of methanol. The maximum theoretical amount of residual methanol is 0.5 wt% relative to the mass of the adhesive. However, the curing temperature is 10 °C higher that the boiling point of methanol (65 °C) resulting in small amounts present within the formulation.

**Fig. 1:**
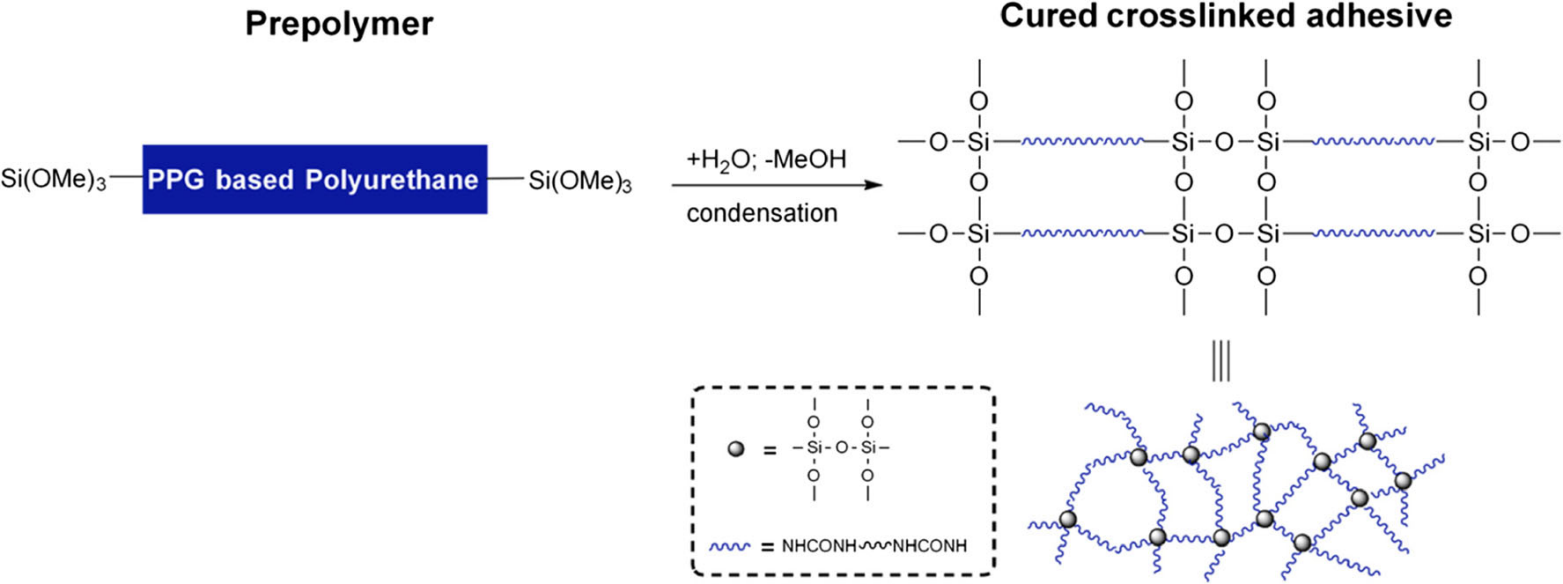
Mechanism of polymer matrix formation

The PSA formulation can be loaded with an API and appropriate excipients by solubilization prior to cure to give homogeneous PSA films, Fig. 1. No additional volatile solvent is added in any of stage of this process.

As ibuprofen is present in the PSA during curing, it is important to investigate any influence it may have on the curing process. Namely, the formation of by-products of ibuprofen and PPG-PU and stability of the API under the curing conditions. In order to verify that ibuprofen (wt% between 10 and 20) does not interact with the adhesive in an irreversible way and/or degrade during the PSA cure, simple mass balance calculations were employed. The method is based on a comparison of theoretical and actual mass fractions of ibuprofen in fabricated patches. A theoretical dose of ibuprofen in patches (D_th_) can be estimated using eq. 1.

1 $$ {D}_{\mathrm{th}.}=\frac{M_s\times {F}_{\mathrm{API}}}{A} $$

where *D*_th._—theoretical dose per cm^2^, *M*_*s*_—mass of sample, *F*_API_—mass fraction of API in formulation, *A*—area of sample in cm^2^.

An actual dose (*D*_*a*_) was determined by submerging samples into 10 mL of acetonitrile/water mixture for 24 h, which was then assayed using the validated HPLC method for ibuprofen. The obtained results for three ibuprofen formulations are given in Table 2. An average of 12 measurements for each formulation was accepted as a statistically reliable value. A relative standard deviation of 10% was taken into account as an acceptance criterion according to the European Pharmacopoeia.

**Table 2:** Comparison of theoretical and actual doses of ibuprofen in TEPI® patches

Parameter	D_th._, mg cm^−2^			D_a_ [HPLC], mg cm^−2^		
API, wt%	10	15	20	10	15	20
S1	1.63	2.67	3.30	1.52	2.47	3.50
S2	1.71	2.25	3.43	1.66	2.27	3.26
S3	1.62	2.25	3.03	1.83	2.20	3.16
S4	1.35	2.37	2.76	1.51	2.30	2.84
S5	1.42	1.97	2.98	1.40	2.07	3.04
S6	1.34	2.25	3.82	1.40	2.12	3.62
S7	1.38	2.22	2.93	1.46	2.16	2.98
S8	1.62	2.14	3.37	1.56	2.09	3.20
S9	1.73	2.46	3.11	1.77	2.28	3.03
S10	1.54	2.25	2.97	1.56	2.17	3.10
S11	1.61	2.49	3.08	1.61	2.52	3.07
S12	1.45	2.49	3.61	1.45	2.46	3.65
Average	1.53 ± 0.14	2.32 ± 0.19	3.20 ± 0.31	1.56 ± 0.14	2.26 ± 0.15	3.20 ± 0.26
Relative standard deviation, %				8.79	6.80	8.04
Mean recovery from theoretical, %				102	98	100

**Fig. 2:**
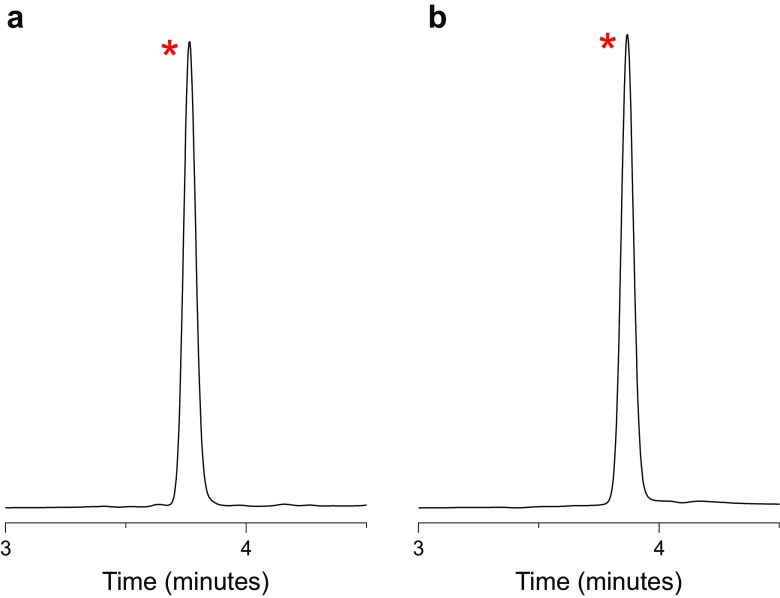
Expanded HPLC traces. **a** *Ibuprofen extracted from cured patches. **b** *Representative peak of pure ibuprofen

The recovery values show that the mass balance of ibuprofen is conserved within the analytical error margin. The obtained HPLC traces correspond to the mass balance evaluation revealing no additional peaks (Fig. 2). Therefore, ibuprofen does not form side products with the reactive PPG-PU and/or degrade during the cure process.

Further analysis on the fabricated patches with various wt% of ibuprofen included uniformity of the product, permeation using a rate-limiting membrane, adhesion, and market requirements. Examples can be found in Figs. S1 and S2 and Table S1. However, for the final formulation, 10 wt% of the API was employed to simulate existing commercial products and to ensure a local pain treatment other than a systemic delivery.

### API Release from the Patch

The drug release characteristics of the basic formulation (no added excipients) were studied in vitro using artificial non-rate-limiting membranes. This study allows for confirmation that the cured adhesive is a reservoir for ibuprofen and does not limit its use as a transdermal drug delivery system. These experiments can be performed using non-rate-limiting membranes, including porous silicone, nylon, or PTFE. In this study, nylon was employed as the test model membrane for a passive release. The studied formulation contained 10 wt% of API (patch thickness 160 μm) without adding any permeation enhancers. Patches are approximately 12 × 8.5 cm with 10 mm corner fillet radius (101 cm^2^). A mixture of DEGEE:PBS (10:90) was employed as the acceptor solution and the temperature maintained at 32 ± 1 °C. Samples were taken periodically for a total period of 24 h. It must be noted that hydrophobic drugs such as ibuprofen have a limited solubility in aqueous media (e.g., PBS buffer), which limits the flux by a quick saturation of the acceptor solution. Hence, a small amount of DEGEE (10%) solution to help circumvent this saturation issue.

**Fig. 3:**
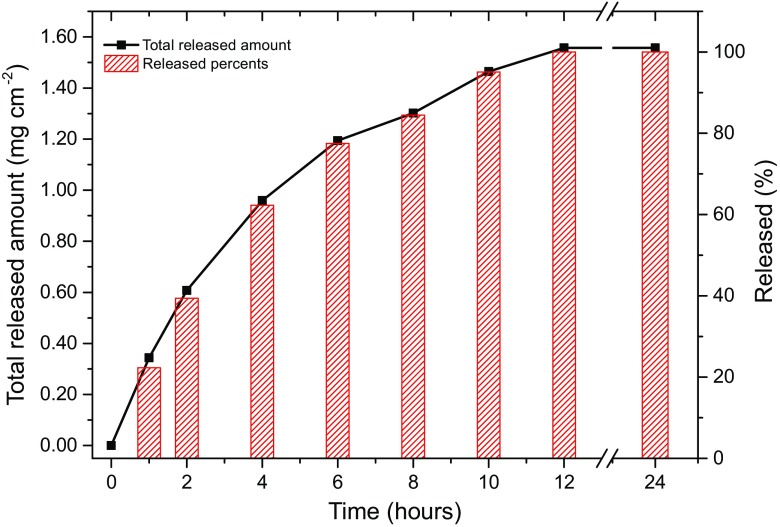
Release of ibuprofen from 10 wt% patches. A mixture of PBS/Transcutol (90/10 *v*/*v*) was used as the acceptor solution

The data depicted in Fig. 3 clearly shows that there is no physicochemical barrier for ibuprofen being released from the patch. A total of 1.5 mg cm^−2^ of ibuprofen was released from the patch over 12 h, which is equal to the applied dose. Thus, ibuprofen can be released from TEPI® patches which make them excellent candidates for a PSA reservoir for a transdermal drug delivery system.

### API Permeation

#### Comparison of Synthetic Membranes and Human Skin

**Fig. 4:**
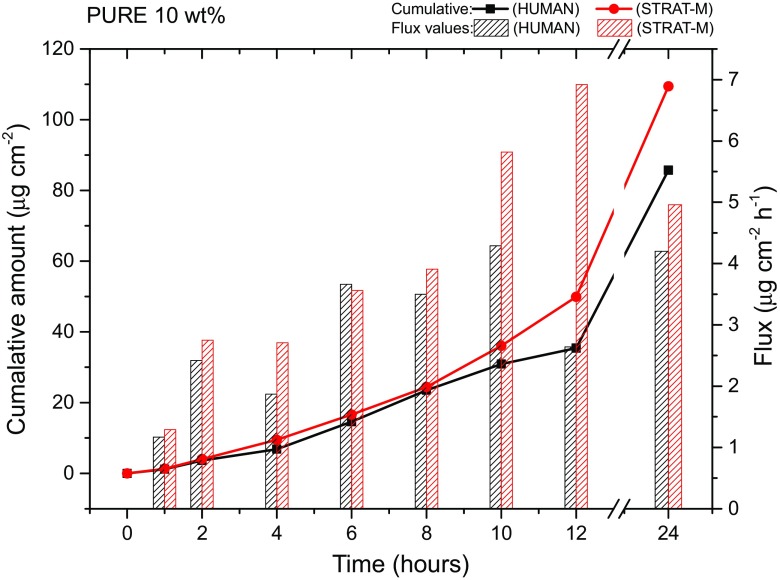
Human skin and Strat-M permeation profiles for ibuprofen 10 wt%, 160 μm patch thickness

Initial studies for assessing ibuprofen permeability employed Merck Millipore Strat-M, a synthetic non-animal-based model for predictive diffusion in human skin. There is a correlation tool on a dedicated website allowing a comparison with human skin for selected APIs, indicating that permeation profiles can vary depending on the drug. However, there is no correlation data available for ibuprofen. Thus, a comparison on the permeation rates of the basic formulation (10 wt% API, 160 μm thickness) across Strat-M and human skin was conducted. The cumulative permeated amount for Strat-M after 24 h was found to be 109.47 μg cm^−2^, whereas for human skin was 85.73 μg cm^−2^ for the same period (Table 3, Fig. 4). Both membranes resulted in similar flux value in 24 h (~ 4 μg cm^−2^ h^−1^) (Table 3). It was therefore concluded that Stat-M shows some correlation for the pure formulation. Interestingly, in the presence of excipients, there is no correlation between the two different membranes. Two formulations including F1 mixtures of glycol (DEGEE:PG = 5:5) and F2 mixtures of glycols with fatty compounds (DEGEE:PG:OD = 5:5:3) resulted in almost seven times higher cumulative amounts and approximately five times higher flux values for Strat-M when compared with human skin (Figs. S3 and S4). Thus, for all the following studies, human skin was employed as the studied membrane to ensure credibility of the results.

**Table 3:** Comparison of Strat-M and human skin on permeation rates of ibuprofen

Membrane	API (wt%)	Formulation (wt%)	Permeated amount in 24 h (μg cm^−2^)	Flux in 24 h (μg cm^−2^ h^−1^)
Strat-M	10	Basic^*^	109.47	4.96
		F1	584.61	20.06
		F2	883.87	29.31
Human skin		Basic^*^	85.73	4.20
		F1	81.21	3.78
		F2	118.61	5.93

^*^No excipients were employed for the basic formulation. However, 2 wt% of BA was employed in both cases to enhance the API solubility in the adhesive matrix

#### Optimized Formulation and Impact of Excipients on Patch Adhesion

Skin permeability is a complex phenomenon depending on chemical and physical properties of each API. For instance, transdermal delivery of nicotine achieves a therapeutic concentration in blood plasma, while ibuprofen topical formulations (gels, creams etc.) are used for a local pain treatment only. In many cases, in order to help overcome the skin barrier and deliver a required amount of API, formulations are enhanced with excipients. For example, commercially available ibuprofen gels/creams usually contain alcohols (ethanol or isopropanol) which facilitate an effective API permeation through the skin. As it can be seen from Figs. 3 and 4, the release and permeation profiles are very different for the ibuprofen patch which can be explained by the skin barrier. There are many permeation enhancers reported in literature, which have different mechanisms of action [30]. Interestingly, commonly employed glycols have been reported to show synergistic actions when combined with fatty compounds which allows for a higher flux of, mainly hydrophobic, APIs across a studied membrane. To exemplify the possibility to employ these mixtures with the TEPI® drug delivery system, we prepared ibuprofen patches with various excipient formulations keeping any the other variables constant (10 wt% API, 160 μm thickness). The basic formulation (no excipients) was employed for a comparison.

**Fig. 5:**
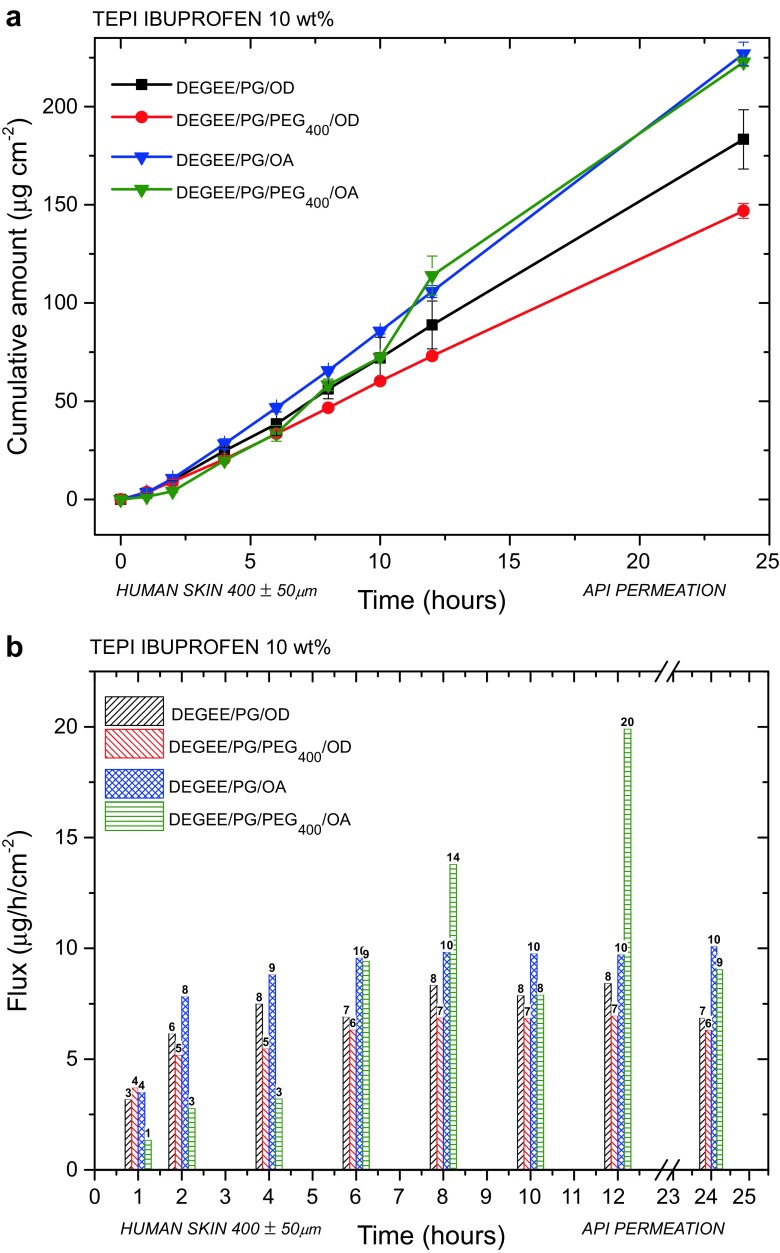
**a** Cumulative amounts and **b** flux values for ibuprofen 10 wt% across human skin (400 ± 50 μm) utilizing mixtures of glycols and fatty compounds over a 24-h period

Commonly employed glycol mixtures (5:5 wt%) including DEGEE, PG, PEG_400_ combined with fatty compounds were investigated mainly due to their chemical properties and low toxicity. Octadecanol and oleic acid were employed as the fatty compounds. Oleic acid is one of the leading penetration enhancers used for transdermal applications, and as a fatty compound, it can act synergistically with glycols, when it is employed in small concentrations, to increase the flux of lipophilic APIs. Detailed mechanistic studies have revealed that oleic acid pools in the stratum corneum increases bilayer fluidity. The results for these combinations can be found in Table 4 and Fig. 5. A detailed summary of the formulations employed can be found in Table S2). Combination of various glycols with oleic acid resulted in higher flux values if compared to the equivalent mixtures with octadecanol. Both formulations including oleic acid resulted in very similar permeated amounts (> 220 μg cm^−2^) over a 24 h period. However, the formulation without PEG_400_ showed constant flux values over the total time period of the study, and thus, we decided that the best formulation for ibuprofen 10 wt% is DEGEE: PG: OD (5:5:1.5).

**Table 4:** Impact of enhancers on the drug delivery performance of TEPI ibuprofen 10 wt% patches

No	Formulation (wt%)	Permeated amount in 24 h (μg cm^−2^)	Flux in 24 h (μg cm^−2^ h^−1^)	Delivered in 24 h (%)
F3	Basic (no excipients)	85.73	4.20	5.4
F4	DEGEE: PG: OD (5:5:1.5)	183.35	6.86	11.2
F5	DEGEE: PG: PEG_400_: OD (5:5:2.5:1.5)	146.95	6.32	8.1
F6	DEGEE: PG: OA (5:5:1.5)	226.90	10.09	17.3
F7	DEGEE: PG: PEG_400_: OA (5:5:2.5:1.5)	222.65	9.06	13.6

**Fig. 6:**
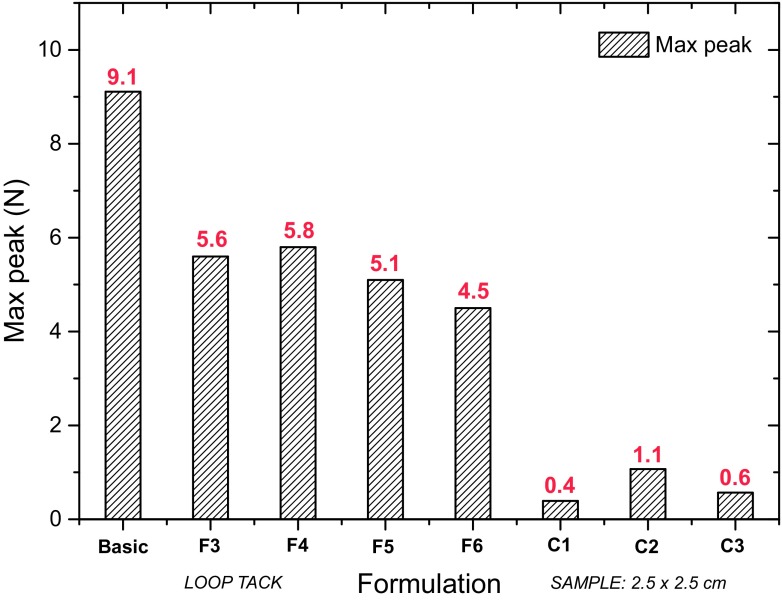
Comparison of loop tack adhesion of TEPI ibuprofen 10 wt% and commercially available products

An important characteristic of any transdermal patch is effective adhesion. An overall drug delivery performance of a patch depends on its area, and therefore, a detached surface will reduce the amount of API permeated across the skin. This becomes more pronounced when a patch needs to be worn for a long period of time, e.g., 7 days. To overcome these issues, a patch should strongly adhere to the skin over the entire period of application. Therefore, the impact of the various excipient mixtures on the adhesion of the resulting patch was assessed (Fig. 6). The adhesion of the pure formulation showed (as expected) the highest adhesion (~ 9 N). Upon addition of excipients, the adhesion dropped approximately two times (~ 5 N) but maintained within a similar range for all studied formulations (4.5–5.8 N). Commercially available patches were employed as a comparison. Figure 6 illustrates that TEPI patches have a significantly higher adhesion in comparison to patches currently available in the UK market. It must be noted that the high adhesion values obtained for the TEPI patches still allow for a painless removal and considered to be within an acceptable range if compared to commercially available adhesive medical tapes who resulted in a maximum peak ~ 4 N.

#### Stability Study for the Optimized Formulation

**Fig. 7:**
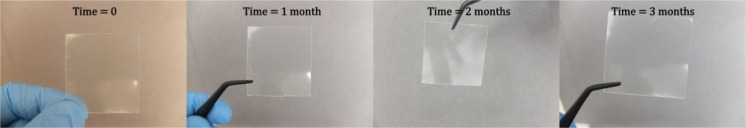
Images illustrating the stability of TEPI® ibuprofen patches

A short-term stability study (3 months) for the optimized formulation was also performed to assess the quality and estimate the shelf-life of the resulting patch. Patches are approximately 12 × 8.5 cm with 10 mm corner fillet radius (101 cm^2^). This study was outsourced and took place under Good Laboratory Practice, GLP conditions (40 °C and 70% relative humidity). Storage under these conditions showed that ibuprofen transdermal patches based on TEPI® technology are stable. Samples were evaluated for crystallinity of the API and determination of the dose by an extraction method utilizing validated HPLC methods. Formation of ibuprofen crystals was not observed and equal amounts of ibuprofen were extracted from the patches after 1, 2, and 3 months of storage (Fig. 7, Table 5, Fig. S5).

**Table 5:** Stability study of the optimized formulation under GLP conditions at 40 °C and 70% RH

Time (months)	Extracted mass, μg	Extracted mass per area, μg cm^−2^
0	1240	2480
1	1230	2460
2	1256	2512
3	1229	2458
Average	1238.75	2477.5
RSD, %	1.01	1.01

## Conclusions

In this study, the use of novel, solvent-free transdermal drug delivery system has been investigated. TEPI® system can be utilized as a drug reservoir for ibuprofen allowing for control of the API dose per area within analytical error margin. It was found that ibuprofen can easily be released from fabricated patches and penetrate through human skin membranes. The flux of API across the membranes can be controlled by including permeation enhancers. The adhesion of TEPI ibuprofen patches was also assessed and it was significantly higher if compared to products available on the market. Interestingly, although the presence of enhancers in our formulations had a significant impact on the adhesion, the resulting patches had notably high adhesion values when compared to commercial products. Finally, initial studies on the stability of the optimized formulation suggested that the ibuprofen patch is stable under accelerated temperatures and no change in the physical characteristics has been observed.

## Electronic supplementary material

ESM 1 (DOCX 211 kb)
