# Supplementary material for: Transdermal Delivery of Ibuprofen Utilizing a Novel Solvent-Free Pressure-sensitive Adhesive (PSA): TEPI® Technology
Source: J Pharm Innov. 2017 Dec 14;13(1):48–57. doi: 10.1007/s12247-017-9305-x (PMC5816128; doi:10.1007/s12247-017-9305-x)
Supplement: Supplementary file 1 — (DOCX 211 kb) [file 12247_2017_9305_MOESM1_ESM.docx]

**Supporting Information**


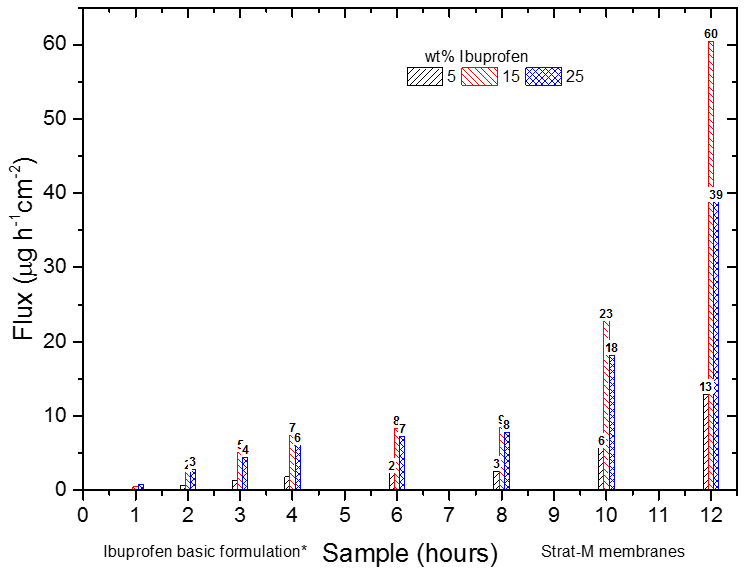


**Figure S1.** Effect of various ibuprofen wt% on cumulative amounts (up) and flux values (down) across a rate limiting membrane.

**Table S1.** Effect of various ibuprofen wt% on cumulative amounts and flux values across a rate limiting membrane.

| No | Basic formulation  (wt%) | Dose applied  (mg per patch)^*^ | Permeated amount  in 12 h  (μg cm^-2^) | Flux  in 12 h  (μg cm^-2^ h^-1^) |
| --- | --- | --- | --- | --- |
| 1 | 5 | 80 | 27.36 | 12.93 |
| 2 | 15 | 240 | 94.40 | 60.47 |
| 3 | 25 | 400 | 86.22 | 38.86 |

^*^Patches are approximately 12 cm × 8.5 cm with 10 mm corner fillet radius (101 cm^2^).

**Figure S2.** Effect of various ibuprofen wt% on the patch adhesion (loop tack).

**Figure S3.** Comparison of Strat-M membrane and excised human skin for the formulation F1 (DEGEE:PG = 5:5), ibuprofen 10wt%.

**Figure S4.** Comparison of Strat-M membrane and excised human skin for the formulation F2 (DEGEE:PG:OD = 5:5:3), ibuprofen 10wt%.

**Table S2.** Summary of the studied formulations.

| **N_o_** | **Adhesive**  wt% | **Titanium**  ***n*-butoxide**  wt% | **API**  wt% | **BA**  wt% | **DEGEE**  wt% | **PG**  wt% | **PEG_400_**  wt% | **OD**  wt% | **OA**  wt% |
| --- | --- | --- | --- | --- | --- | --- | --- | --- | --- |
| **F4** | 75 | 1.5 | 10 | 2 | 5 | 5 |  | 1.5 |  |
| **F5** | 72.5 | 1.5 | 10 | 2 | 5 | 5 | 2.5 | 1.5 |  |
| **F6** | 75 | 1.5 | 10 | 2 | 5 | 5 |  |  | 1.5 |
| **F7** | 72.5 | 1.5 | 10 | 2 | 5 | 5 | 2.5 |  | 1.5 |

**Figure S5**. HPLC traces of the ibuprofen patches after 0 (day 1), 1, 2 and 3 months of storage.
